# Monitoring Crop Status in the Continental United States Using the SMAP Level-4 Carbon Product

**DOI:** 10.3389/fdata.2020.597720

**Published:** 2021-01-18

**Authors:** Patrick M. Wurster, Marco Maneta, John S. Kimball, K. Arthur Endsley, Santiago Beguería

**Affiliations:** ^1^ Regional Hydrology Lab, Geosciences Department, University of Montana, Missoula, MT, United States; ^2^ Department of Ecosystem and Conservation Sciences, W.A. Franke College of Forestry and Conservation, University of Montana, Missoula, MT, United States; ^3^ Numerical Terradynamic Simulation Group, University of Montana, W.A. Franke College of Forestry and Conservation, Missoula, MT, United States; ^4^ Estación Experimental de Aula Dei, Consejo Superior de Investigaciones Científicas (EEAD-CSIC), Zaragoza, Spain

**Keywords:** agriculture, drought, crop yield, l4C, SMAP, crop condition, GPP

## Abstract

Accurate monitoring of crop condition is critical to detect anomalies that may threaten the economic viability of agriculture and to understand how crops respond to climatic variability. Retrievals of soil moisture and vegetation information from satellite-based remote-sensing products offer an opportunity for continuous and affordable crop condition monitoring. This study compared weekly anomalies in accumulated gross primary production (GPP) from the SMAP Level-4 Carbon (L4C) product to anomalies calculated from a state-scale weekly crop condition index (CCI) and also to crop yield anomalies calculated from county-level yield data reported at the end of the season. We focused on barley, spring wheat, corn, and soybeans cultivated in the continental United States from 2000 to 2018. We found that consistencies between SMAP L4C GPP anomalies and both crop condition and yield anomalies increased as crops developed from the emergence stage (r: 0.4–0.7) and matured (r: 0.6–0.9) and that the agreement was better in drier regions (r: 0.4–0.9) than in wetter regions (r: −0.8–0.4). The L4C provides weekly GPP estimates at a 1-km scale, permitting the evaluation and tracking of anomalies in crop status at higher spatial detail than metrics based on the state-level CCI or county-level crop yields. We demonstrate that the L4C GPP product can be used operationally to monitor crop condition with the potential to become an important tool to inform decision-making and research.

## 1. Introduction

Accurate crop condition assessments provide valuable information to farmers and policy-makers regarding the security and economic viability of agriculture. Farmers consider crop condition when determining where to best allocate costly inputs or to identify land management practices that maximize yields and profits. Policy-makers consider crop condition when determining where to allocate limited resources or to identify when and where safety nets are most needed during times of crisis. Crop condition assessments are considered along with demand expectations to forecast crop prices, which directly affect farm income and agricultural market volatility (Lehecka, 2014). Crop condition is often a reflection of the prevailing climatic conditions. However, changes in crop condition due to climatic variability vary by crop type, farming practices, and over time with crop phenology (Zipper et al., 2016; Peña-Gallardo et al., 2019; Wurster et al., 2020). For example, cereal crops tend to be more resilient to drought than legumes (Daryanto et al., 2017). The condition of irrigated crops may decline due to pluvial events while the status of rain-fed crops may improve (Doughty et al., 2018). Cereal crops under higher temperatures may improve at the beginning of the growing season but may degrade near the end of the season (Klink et al., 2014). Therefore, accurately describing crop conditions at spatial and temporal scales meaningful to decision-makers is important for early detection of potential issues but also poses a technical challenge.

Several climate indices have been developed and used to determine the impact of climatic variability on agriculture. The Palmer Drought Severity Index (PDSI) is based on temperature, precipitation, runoff, soil moisture, and atmospheric water demand Palmer (1965). The Standardized Precipitation Index (SPI) is based on precipitation alone (McKee et al., 1993). The Standardized Precipitation Evapotranspiration Index (SPEI) uses a similar statistical framework as the SPI but considers precipitation minus potential evapotranspiration (Vicente-Serrano et al., 2010). The Evaporative Demand Drought Index uses potential evapotranspiration alone to describe anomalies in atmospheric water demand (Hobbins et al., 2016). Climate indices have been used in several different studies to infer the relationship between meteorological anomalies to crop production or yield over the growing season and at different time-scales (Meyer et al., 1991; Zipper et al., 2016; Peña-Gallardo et al., 2019; Wurster et al., 2020). These studies provide insight into the timing, severity, and duration of climatic anomalies that, to differing extents, drive anomalies in crop production or yield, but with several limitations. Focusing on the crop production or yield response to climatic variability does not capture within-season changes in crop condition, as crop production and yield data are only available at the end of each season. Further, currently available climate indices either do not consider soil moisture (e.g., SPI, SPEI, and EDDI) or rely on water-balance models based on meteorological conditions to estimate soil moisture indirectly (e.g., PDSI). Soil moisture is arguably one of the most critical drivers of crop condition because agricultural drought is initiated by soil moisture deficits (Mishra and Singh, 2010).

For over 40 years, vegetation indices derived from global satellite remote sensing observations have provided some insights into crop condition over the course of the growing season. The Normalized Difference Vegetation Index, or NDVI (Rouse et al., 1974; Tucker, 1979), is commonly used to study the impacts of climatic anomalies on crop condition by relating changes in NDVI values to SPI (Ji and Peters, 2003) or SPEI (Xu et al., 2018). Satellite-based remote sensing techniques have also utilized information from both optical and thermal spectral wavelengths to infer soil moisture conditions over cultivated areas (Nemani et al., 1993; Mallick et al., 2009; Amani et al., 2016). More recently, satellite observations of solar-induced canopy fluorescence (SIF) have been found to be effective proxies for cropland productivity, albeit at relatively coarse (≥0.5 degree) spatial resolution (Guanter et al., 2014; Guan et al., 2017). In particular, the application of satellite remote sensing for agricultural monitoring is constrained by trade-offs between optimizing finer spatial resolution for delineating heterogeneous croplands and frequent temporal sampling for monitoring dynamic crop stage development. Satellite images of the land surface may be obscured by cloud cover or distorted by aerosols in the atmosphere. While vegetation indices like NDVI have been found to be strongly correlated with measured soil moisture conditions over homogeneous vegetation covers (Gu et al., 2008), the soil moisture measurements available for comparison were widely distributed spatially and did not fully capture soil heterogeneity or the myriad of different soil types. NDVI is also influenced by the soil background (Fensholt et al., 2006), which can be particularly problematic for croplands in the early season where bare soil is visible.

Remote sensing developments have also resulted in models describing the gross primary production (GPP) of crops. GPP is a key metric for agricultural production because it is proportional to the crop biomass accumulated through fixation of atmospheric carbon over the growing season (Beer et al., 2010). Satellite-based observations of GPP, in conjunction with a growing distribution of a global system of eddy covariance flux towers, have resulted in a variety of methods for estimating GPP (Running et al., 2004; Rahman et al., 2005; Jones et al., 2017). One common method is the light-use efficiency model (Monteith, 1972; Hilker et al., 2008), which defines GPP as the product of the available photosynthetically active radiation (PAR), the fraction of PAR absorbed by vegetation (fPAR), and the efficiency (ϵ) at which the vegetation utilizes the absorbed solar energy to produce organic matter from net photosynthesis ($\text{gC}\;{\text{MJ}}^{-1}$). *e* is limited by environmental constraints including unfavorable temperature, atmospheric vapor pressure deficit, and plant-available soil moisture (Hilker et al., 2008). ϵ has been calibrated for different plant functional types (PFTs) on the basis that the various plant community types populating a given biome have evolved a similar collective canopy response to climatic variability (Gower et al., 1999).

In these remote-sensing-based and model-based approaches to estimate carbon exchange between land and atmosphere, available soil moisture has been identified as a critical variable (Pastor and Post, 1986; Seneviratne et al., 2010; He et al., 2014). Remote sensing methods for monitoring soil moisture are not new (e.g., Njoku and Entekhabi (1996)). However, data on soil moisture conditions at regional or global scales, with adequate frequency for monitoring GPP, have historically been unavailable (Hilker et al., 2008). The NASA Soil Moisture Active Passive (SMAP) mission was launched in January, 2015, and aimed to resolve the soil moisture constraint on land-atmosphere carbon (${\text{CO}}_{2}$) exchange, including GPP (Jones et al., 2017). The SMAP sensor employs a low frequency (L-band) microwave radiometer providing global 1–3 day repeat sampling of L-band (1.4 GHz) brightness temperatures (Tb) with enhanced sensitivity to soil moisture within the surface (0–5 cm depth) soil layer (Entekhabi et al., 2010). The SMAP mission includes operational production of model enhanced Level-4 soil moisture (L4SM) and carbon (L4C) products that benefit from the land model assimilation of SMAP Tb retrievals and other observations to produce continuous global daily estimates of surface and root zone (0–1 m depth) soil moisture and soil temperature (Reichle et al., 2016); the L4SM results are used with satellite (MODIS) vegetation observations and other ancillary inputs to derive L4C daily carbon fluxes, including GPP (Jones et al., 2017). The SMAP L4C GPP product provides a potential opportunity to monitor cropland productivity globally and with daily temporal fidelity. The L4C GPP estimates include the influence of atmosphere and soil-moisture related restrictions on productivity and PFT-specific calibrations for broadleaf and cereal crop types. However, the L4C utility in cultivated areas and sensitivity to different crop phenological stages have not been established and are needed to clarify its potential to inform agriculture-related decision-making by stakeholders outside of the scientific community (Sanders and Masri, 2016).

Crop condition and crop progress reports, in contrast to remote-sensing-based GPP estimates, are widely available for many important crops in the US. Farmers and other agricultural experts conduct voluntary field assessments of crop condition in relation to expected yield and assessments of crop progress at key phenology stages. The assessments are reported to the US Department of Agriculture and made available by the National Agricultural Statistics Service (NASS). Reports on crop condition and crop progress are conducted weekly and made available at the start of the following week. These crop condition rankings were recently transformed by Begueria and Maneta (2020) into a continuous crop condition index (CCI) that describes weekly crop condition at the state level. The CCI has demonstrated the ability to predict crop yields weeks before the actual yields are realized (Begueria and Maneta, 2020). While the CCI offers a continuous metric describing crop condition at high temporal scales (weekly), the relatively coarse state-wide assessment may not capture the heterogeneity of crop conditions, particularly in larger states where management systems, climate or weather conditions over cultivated areas, and associated crop yields may vary considerably.

In this study, we combined the information in the satellite remote-sensing-based SMAP L4C GPP product with the CCI to evaluate whether the accumulated GPP anomalies provide a meaningful metric of crop conditions for the major cereal and broadleaf crop types within the continental US. We also compared the L4C accumulated GPP anomalies to anomalies in reported crop yield. Our objectives were to (1) determine the correlations between GPP anomalies estimated by the L4C and weekly crop condition anomalies at the state level, (2) determine the correlations between GPP anomalies and annual crop yield anomalies at the county level, and (3) inspect the variables driving the L4C GPP model and compare them to the actual biophysical drivers of crop GPP. This analysis provides a comparison of GPP anomalies estimated by a remotely sensed, soil moisture-driven carbon flux model to independent state-level assessments of weekly crop condition and reported county-level crop yield surveys.

We hypothesized that annual variability in GPP at a particular time (week) during the growing season has a strong association with the observed crop conditions at that time. We also posited that GPP anomalies are increasingly indicative of end-of-season crop yields as the growing season progresses. At the state level, we compared anomalies in GPP to anomalies in CCI in each week over the growing season from 2000 to 2018. At the county level, we compared county-averaged GPP anomalies in each week to annual crop yield anomalies during the same period. We assumed that the negative (positive) anomalies in weekly crop condition and yield would correspond to negative (positive) anomalies in GPP of similar relative magnitude.

## 2. Methods

### 2.1. Data

#### 2.1.1. Crop Information

Both crop condition surveys and crop yield surveys were obtained from the USDA National Agricultural Statistics Service (NASS). The crop condition surveys are conducted weekly during the growing season via visual assessments by agricultural experts and are reported at the state level. The surveys report the percentage of planted area categorized as follows: “Very Poor” (near to complete crop failure), “Poor” (heavy losses), “Fair” (less than normal yield), “Good” (normal yield), and “Excellent” (above normal yield) (USDA, 2020). To overcome the statistical limitations of the ordinal crop condition survey data, Begueria and Maneta (2020) transformed the categorical crop condition metrics into a continuous crop condition index (CCI). The CCI isolated the random variability in crop condition reports from spatial, long-term, and intraseasonal tendencies inherent to the subjective visual assessments. The isolated random effect was assumed to provide an unbiased metric of crop condition, where positive values indicated above-normal crop condition and negative values indicated below-normal crop condition. At the county-level, annual crop yield data was retrieved from the NASS. Phenology data for each crop type was retrieved from the NASS and used to identify the beginning, intermediate, and end stages of the growing season. Phenology data was available at the state-level. A complete list of the different phenological stages for each crop, including definitions, is available from the NASS “Crop Progress/Crop Weather Terms and Definitions”. The key stages used here were “emerged”, “silking”, and “mature” for corn; “emerged”, “blooming”, “dropping leaves”, and “harvested” for soybeans; and “emerged”, “headed”, “coloring”, and “harvested for both barley and spring wheat. Phenology was reported by NASS as the percent of planted area where a crop has developed to a particular phenological stage. We considered that a given crop had progressed to a particular stage on the week in which 50% of the total planted area within a given state was reported as completed. The 50% threshold was chosen because it was apparent that percentages of planted area were already proceeding to the next stage by the week in which a given stage was reported as 100% complete. In some cases, large gaps in the reported percentages were observed moving from one week to the next during a given year (e.g., 30% at one week, 60% at the next week). Further, phenology was not reported for all stages in some states for all years within the period of record. To simplify the timing of each phenology stage for a particular crop in a particular state, the median week across the period of record at which the stage was observed to be closest to 50% completed in each particular state was used.

#### 2.1.2. Soil Moisture Active/Passive Level-4 Carbon

GPP throughout the growing season is obtained from the NASA Soil Moisture Active/Passive (SMAP) Level-4 Carbon (L4C) product (Jones et al., 2017). The L4C product is derived from a satellite data-driven Terrestrial Carbon Flux (TCF) model using MODIS land cover and 8-day canopy fractional photosynthetic active radiation (fPAR), SMAP L4SM daily soil moisture and soil temperature, and daily surface meteorology from the Goddard Earth Observing System 5 Forward Process (GEOS-5 FP) system as primary drivers. The SMAP L4C product provides a complete daily carbon budget, including GPP, heterotrophic respiration, net ecosystem ${\text{CO}}_{2}$ exchange, and the surface soil organic carbon stock. The L4C processing involves a daily time step at 1-km spatial resolution consistent with the MODIS land cover and vegetation inputs. The L4C outputs are posted to a 9-km resolution global grid format, consistent with the SMAP L4SM product, while preserving subgrid spatial averages for up to eight plant function type (PFT) classes within each 9-km grid cell preserved from the finer (1 km) resolution processing. These 1-km subgrid averages were used in this analysis. PFT is defined from the Moderate Resolution Imaging Spectroradiometer (MODIS) MOD12Q1 (Type 5) land-cover classification, which distinguishes up to 8 global PFT classes including cereal and broadleaf crop types (Friedl et al., 2010). In L4C, daily GPP is limited by unfavorable environmental conditions, including low root-zone soil moisture levels, low minimum surface temperature, excessive atmospheric vapor pressure deficit (VPD), and frozen soil conditions. The model framework is detailed in the article by Jones et al., 2017 and summarized below (Eqs 1–3). The L4C response to these limiting environmental conditions is calibrated separately for each PFT based on a global network of eddy covariance ${\text{CO}}_{2}$ flux tower observations.

In this study, two variants of the L4C product were used to obtain GPP: the SMAP L4C operational product (L4C-Ops, version 4) and the L4C Nature Run (L4C-NR, version 7.2). L4C-NR is essentially an offline, model-only version of L4C-Ops. Whereas L4C-Ops uses SMAP L4SM soil temperature and soil moisture estimates informed by the SMAP L-band Tb observations (Reichle et al., 2016; Jones et al., 2017), L4C-NR uses only modeled soil temperature and soil moisture data. By using modeled rather than SMAP-informed soil conditions, L4C-NR is able to run for time periods prior to the launch of SMAP, extending the period of record for terrestrial carbon flux estimates. This also requires the use of different meteorological driver data. L4C-Ops uses the GEOS-5 FP inputs, whereas L4C-NR uses reanalysis data: the Modern Era Retrospective Reanalysis (MERRA-2; (Gelaro et al., 2017)); both are products of the Global Modeling and Assimilation Office (GMAO) at the NASA Goddard Space Flight Center. MERRA-2 is also used for calibrating L4C, since the flux tower period of record largely predates the launch of SMAP. These two driver datasets have similar dynamics but different climatologies. Besides these different driver datasets, L4C-Ops and L4C-NR use the exact same model logic and parameters. While soil moisture model estimates used by the L4C-NR have been shown to have slightly lower performance than L4SM estimates incorporating SMAP radiometer observations, the difference is not statistically significant (Reichle et al., 2017). Both products provide GPP estimates for up to 8 different PFTs, including cereal (e.g., barley and spring wheat) and broadleaf (e.g., corn and soybeans) crops and reported in units of $\text{gC}\;{\text{m}}^{-2}\;{\text{d}}^{-1}$.

**FIGURE 1:**
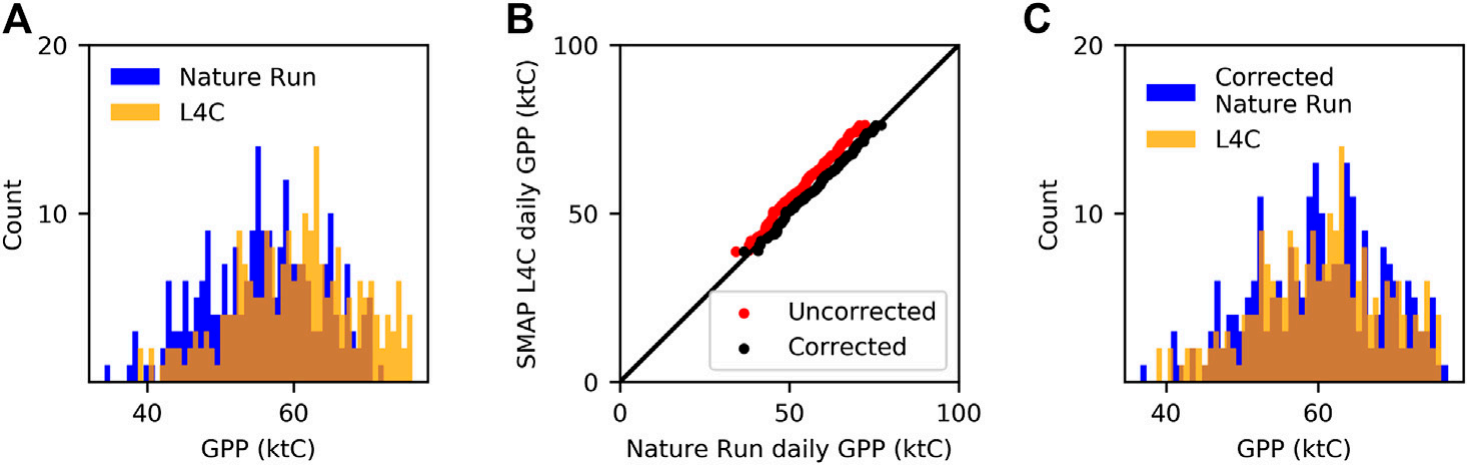
Example of the affine statistical transformation used to minimize the bias between L4C Nature Run compared to L4C Ops. **(A)** Nature Run and L4C Ops GPP values for cereal crops in Colorado. **(B)** Correlations between the Nature Run and L4C Ops before and after the correction. **(C)** L4C Ops and corrected L4C Nature Run values.

The L4C-Ops record begins on March 31, 2015, which provides just 3 years of data for the study period (2000–2018). The relatively short L4C-Ops record represents a challenge for statistical analysis of crop condition and crop yield anomalies. Including L4C-NR in this study provides a much longer period of record. The L4C-NR data are available from 2000 through 2017 and, as a supplement to L4C-Ops, extend the record to a period more suitable for this analysis. Because of the different driver datasets, however, there is a bias difference between the L4C-Ops and L4C-NR datasets when their overlapping periods of record (2015–2017) are compared, which could potentially degrade the quality of L4C-Ops GPP anomalies derived relative to the L4C-NR record. For example, the histograms in Figure 1A illustrate that L4C-NR tended to estimate lower GPP for cereal crops in Colorado than L4C-Ops for the same PFT. To remove this bias, we implemented an affine statistical transformation (see Gudmundsson et al., (2012); Section 2) on the overlapping records, with L4C-Ops being the dependent variable and L4C NR being the independent variable. L4C-Ops estimates and the corresponding L4C-NR estimates were first sorted from smallest to largest, and the regression was then applied to the sorted values. The regression process is shown in Figure 1B. Figure 1C shows that the distribution of the GPP estimates per the corrected L4C-NR was more aligned with the L4C-Ops for cereal crops in Colorado. The regression coefficients produced by the transformation were applied to L4C-NR for the period prior to the L4C-Ops record (prior to March 31, 2015) providing a continuous dataset spanning the 2000-to-2018 study period. Hereafter, the combined L4C-Ops and corrected L4C-NR datasets are referred to as L4C.

### 2.2. Calculations of Anomalies

**FIGURE 2:**
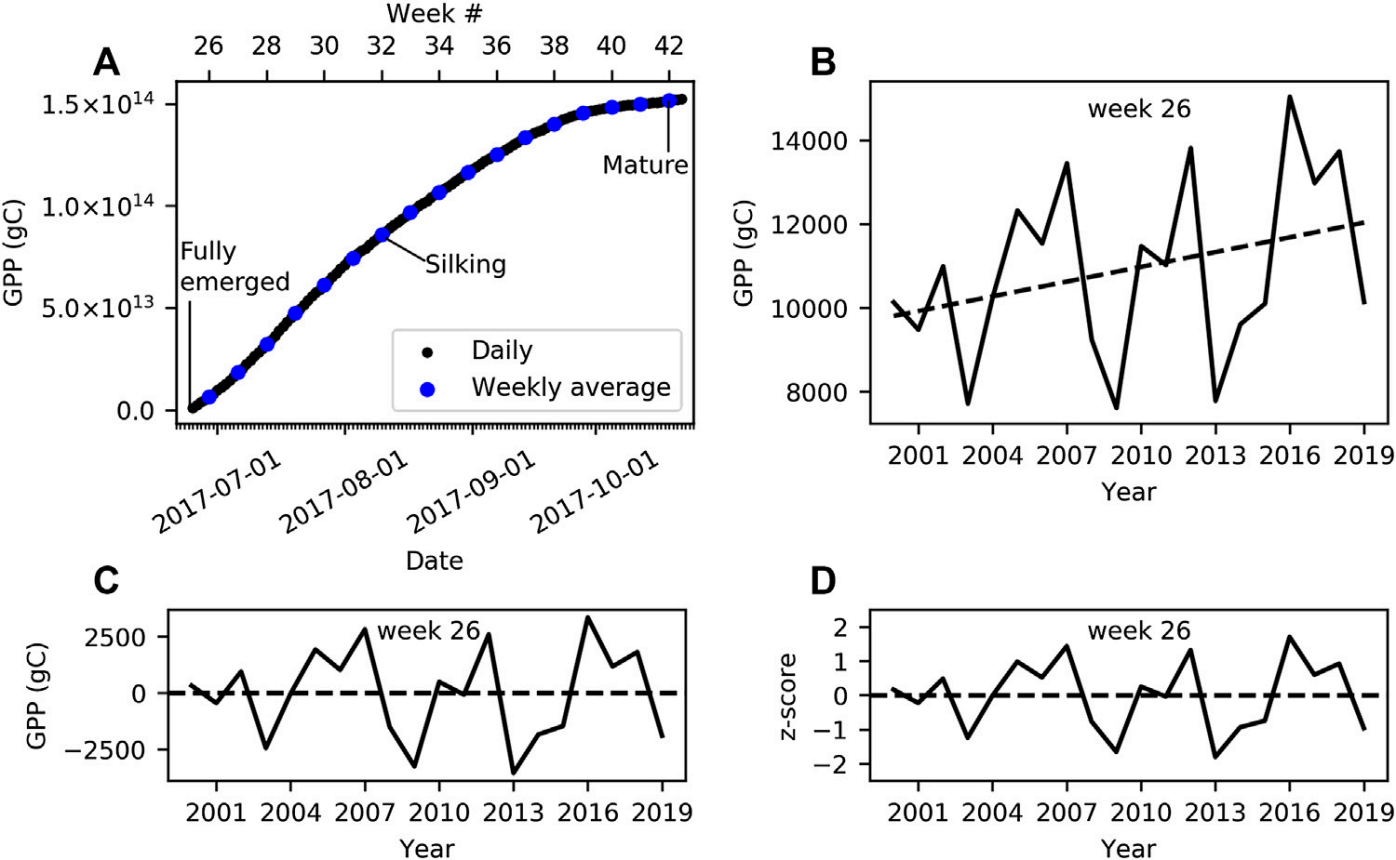
An example of our treatment of the data, in this case corn in Iowa. **(A)** The daily accumulation of total broadleaf GPP over the course of the 2017 season, where the total GPP is the sum of all broadleaf pixels in Iowa over each day. GPP is then accumulated starting when corn has been reported as fully emerged and ending when corn has been reported as mature. **(A)** also shows the weekly average total GPP used in this analysis. **(B)** A time‐series of the total broadleaf GPP at week 26 over the period of record. Notice the positive trend. **(C)** The same time series but with the trend removed using linear regression. **(D)** A time series of z‐scores of the detrended GPP.

L4C GPP cells classified as the cereal PFT were used for comparison of GPP to spring wheat and barley yield and CCI, and L4C GPP cells classified as the broadleaf PFT were used for comparison of GPP to corn and soybean yield and CCI. We assumed that accumulating the total GPP carbon stock (i.e., gC) over the growing season would better capture changes in crop status with respect to environmental stresses/benefits than the daily GPP carbon flux ($\text{gC}\;{\text{m}}^{-2}{\text{d}}^{-1}$) at a discrete point in time. Therefore, the L4C daily values within each 1-km pixel were multiplied by the corresponding spatial coverage ($1000\;{\text{m}}^{2}$) and temporal coverage (1 d) to obtain a total carbon stock (gC). Daily carbon stocks were aggregated to a weekly sum for each cell to produce a weekly carbon stock. Zonal statistics were then performed, within each county for comparison to county-level yield and within each state for comparison to state-level CCI to produce a weekly total GPP value for each week within the growing season. For a given state, the weekly GPP values within that state were accumulated beginning in the median week at which the emerged stage was reported as 50% in the given state and ending in the median week at which the mature/harvested was reported as 50% complete. The process for accumulating county-level GPP was the same; only the phenology weeks were based on the corresponding state’s phenological data. Figure 2A illustrates how the total daily GPP of the broadleaf PFT in Iowa was accumulated over the 2017 corn growing season and averaged by week. Crop yields, and thus GPP, have been increasing over time due to improvements in farming practices and technology (Pingali, 2012) and the influence of rising atmospheric ${\text{CO}}_{2}$ levels (Campbell et al., 2017). Accordingly, positive cropland productivity trends are depicted in both NASS reported crop yields and the L4C GPP record over the study period. To mitigate the influence of the upward trends on the correlation analysis, GPP and CCI were both detrended using linear regression, by week, across the available years (Eq. 4). Figure 2B provides an example of the positive trend over the period of record in accumulated corn GPP in Iowa at week 26. Figure 2C shows the corresponding detrended GPP values. We represent anomalies as z-scores of the detrended GPP, according to Eq. 5. Figure 2D provides an example of corn GPP anomalies in Iowa in week 26 represented as z-scores. The CCI data was treated the same as GPP, where anomalies were represented as the z-scores of weekly detrended CCI values in each state.

The state-level CCI and NASS county-level crop yield anomalies were calculated following the same methodology. Anomalies derived from the L4C GPP record were compared to the CCI and crop yield anomalies in states/counties having at least 15 years of CCI/yield data between 2000 and 2019 using Pearson’s r correlation coefficients. An α level of 0.1 was defined to determine significant correlations, which corresponds to a minimum significant r-value of |0.44| with a minimum sample size of 15 years and a minimum r-value of |0.4| with a maximum sample size of 19 years. The analysis was conducted on each individual week of the growing season across the period of record, although our analysis emphasizes the key phenological stages of the different crop types examined.

### 2.3. Climate, Irrigation, and Model Drivers

To observe how the presence of irrigation impacts the correlations between L4C GPP anomalies and county-level yields, we used irrigated production and total crop production information obtained from the NASS to determine the ratio of irrigated production for each of the four crop types for all counties with available data. We then observed how the correlations change with respect to the areal proportion of agricultural land in irrigated production. Aridity has also been shown to mitigate or enhance the impacts of climatic anomalies such as drought to yield (e.g., (Rhee et al., 2010; Vicente-Serrano et al., 2012), which may impact the observed correlations. Therefore, we used a climate aridity index, calculated as the ratio of annual precipitation to potential evapotranspiration (P/PET) for cultivated areas within each state, to evaluate the influence of climate aridity on the productivity relationships. Here, the aridity index was derived for the study period using 4 km resolution precipitation and potential evapotranspiration data from Gridmet (Abatzoglou, 2013).

We were also interested in determining what climatic variables were the most influential drivers of the L4C GPP anomalies (i.e., 1–3) and how their influence varied spatially. The variation in the climatic drivers and their influence on the L4C GPP estimates were also used to illuminate potential factors explaining the sign and strength of the GPP correlations with the CCI and crop yield anomalies. A multivariate correlation analysis using linear regression was applied to determine the sensitivity of the L4C GPP anomalies to variations in underlying model drivers (Jones et al., 2017), including root-zone soil moisture (RZSM), minimum temperature (Tmin), canopy-absorbed photo-synthetically active radiation (APAR), and vapor pressure deficit (VPD) anomalies. The L4C product includes a diagnostic daily metric ($E_{\text{mult}}$) that defines the total estimated light-use efficiency (and GPP) reduction due to unfavorable environmental conditions. We examined the average growing season $E_{\text{mult}}$ pattern as an additional indicator of climate related constraints on cropland productivity over the CONUS domain. Here, $E_{\text{mult}}$ captures the combined effect of unfavorable daily temperature, VPD, and RZSM conditions on GPP, whereas the above correlation analysis represents the relative influence of APAR and each climate variable on the L4C productivity estimates. The analysis was conducted at the 9-km resolution for all PFT classes across the entire CONUS, with a particular focus on cultivated areas.

## 3. Results

### 3.1. Spatiotemporal Patterns

**FIGURE 3:**
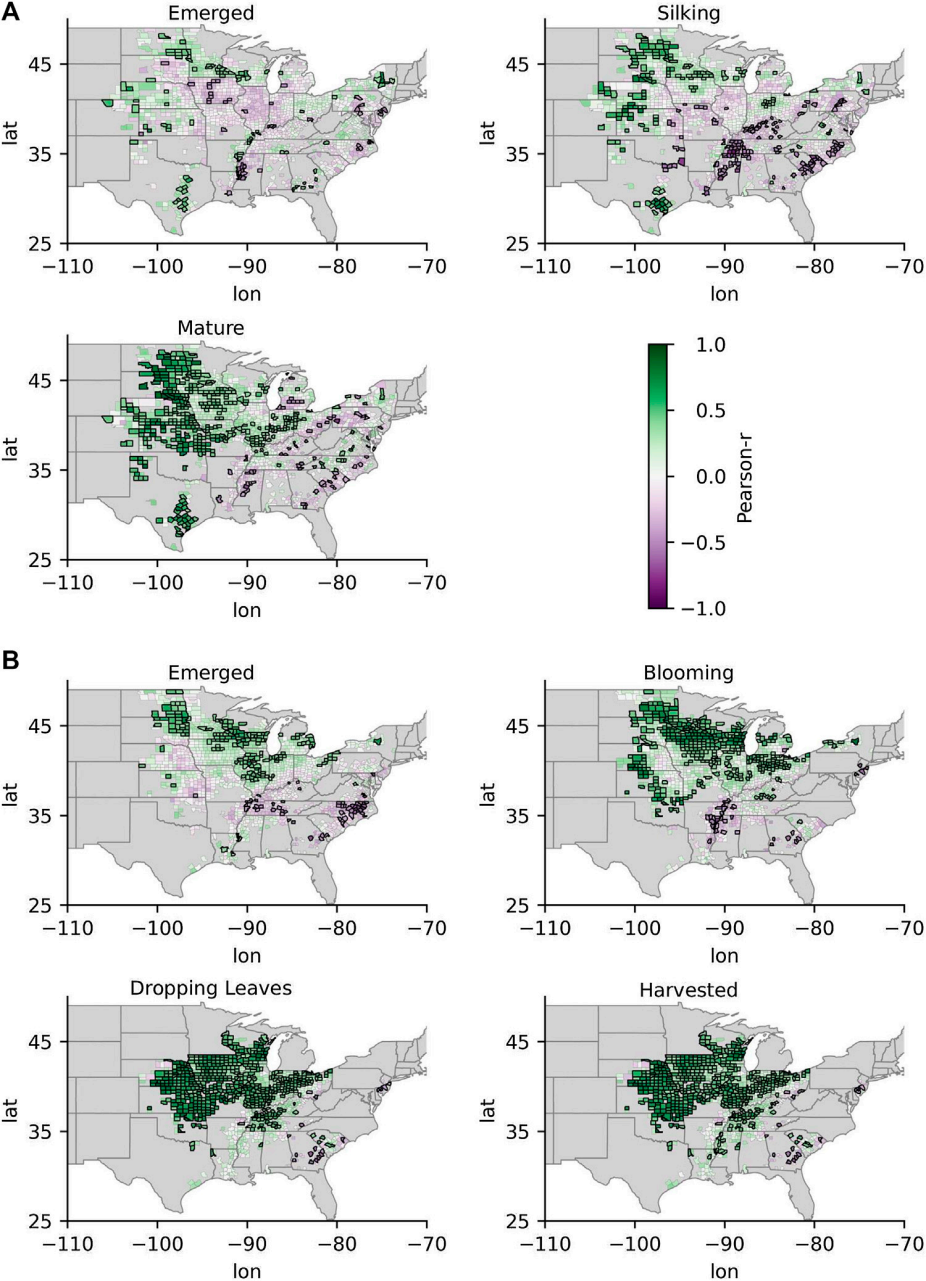
The correlations between GPP anomalies and county-level yield anomalies for the two broadleaf crops corn **(A)** and soybeans **(B)** at critical development stages over the growing season. Darker purples indicate more negative correlations, and darker greens indicate more positive correlations. Counties outlined in black indicate that the correlations were significant. Gray indicates areas where yield data was not available.

**FIGURE 4:**
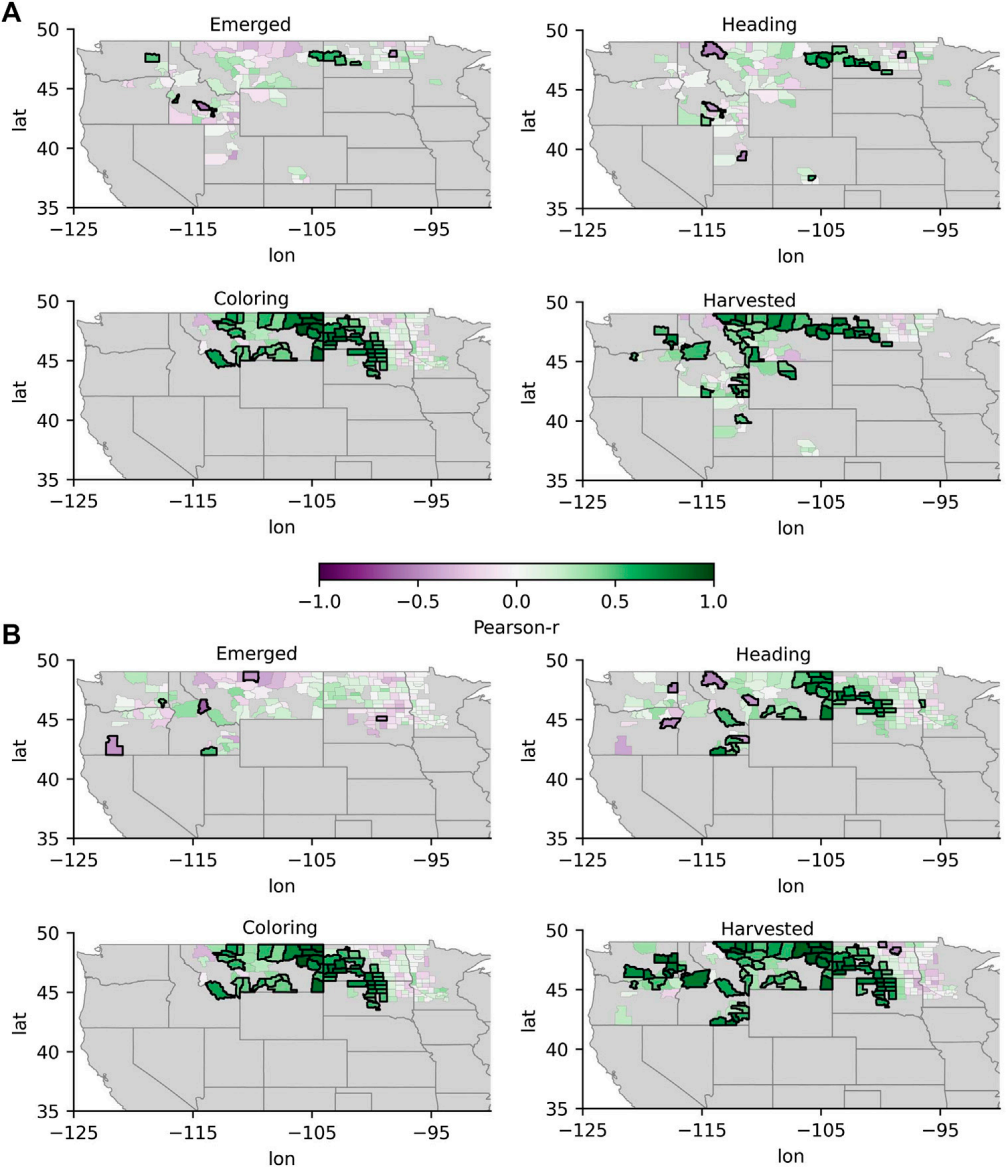
Same as Figure 3, but for the cereal crops barley **(A)** and spring wheat **(B)**.

**FIGURE 5:**
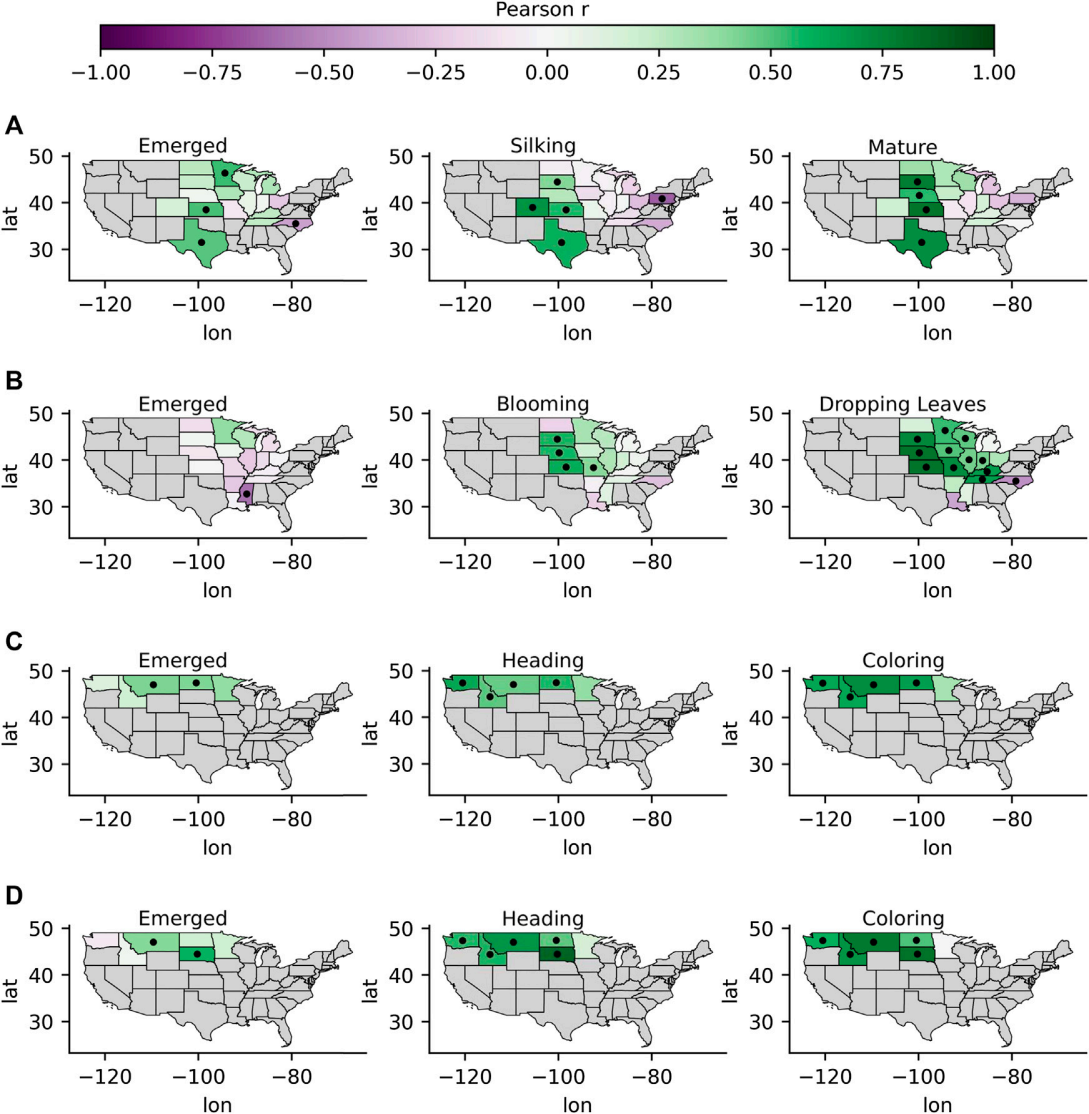
The correlations between GPP anomalies and state‐level CCI anomalies for corn **(A)**, soybeans **(B)**, barley **(C)**, and spring wheat **(D)** at critical development stages over the growing season. Darker purples indicate more negative correlations, and darker greens indicate more positive correlations. The black markers show where correlations were significant. Gray indicates areas where yield data was not available.

The spatiotemporal patterns in the correlations between standardized GPP anomalies and standardized crop yield anomalies at the county-level are shown in Figure 3 for corn and soybeans (broadleaf crops) and in Figure 4 for barley and spring wheat (cereal crops). Spatial patterns in the correlations between standardized GPP anomalies and standardized CCI anomalies at the state-level are shown for all crops in Figure 5. Generally, correlations between GPP anomalies and both yield anomalies and CCI anomalies increased over the progression of phenological stages, with the lowest and highest correlations generally occurring at the emerged stage and the mature stage, respectively. The GPP correlations tended to be stronger in relation to crop yield than with CCI. For the broadleaf crops, soybean CCI and yield tended to have higher correlations with GPP than corn. For the cereal crops, barley and spring wheat produced similar results over both space and time. Presented below is a more in-depth presentation of these results by crop type.

#### 3.1.1. Corn

The emerged stage for corn ranged from week 16 to week 24, or at around late April to mid-June. Patterns were apparent in the spatial distribution of the correlation coefficients of GPP anomalies and both yield anomalies and CCI anomalies, where more positive correlations were observed around the 100th meridian and lower or more negative correlations were observed to the east (Figures 3A,5A). The correlations with state-level CCI at the emerged stage were significant and positive in Texas, Kansas, and Minnesota, but negative and significant in North Carolina. The correlations with county-level yield were significant and positively correlated in 11% of the counties with available yield and phenology data during the emerged stage, with r-values ranging from 0.4–0.7. The highest correlations between GPP and yield were observed in south-east North Dakota, south-east Minnesota, and eastern Texas. Significant and negative correlations with yield were observed in counties around the Missouri, Mississippi, and Ohio Rivers. While we discuss how correlations change in respect to aridity and irrigation in further detail in Section 3.2, it is helpful to note here that irrigated production is dominant around the Missouri, Mississippi, and Ohio Rivers. P/PET values at around the 100th meridian ranged between 0.5 and 0.7, with P/PET values decreasing to the west and increasing to the east. In some cases, GPP anomalies were more strongly correlated with county-level yield than with state-level CCI. For example, the correlations between GPP and yield anomalies were relatively higher in counties in eastern Texas (r: 0.7–0.9), but lower between the Texas state-wide GPP and CCI (r: 0.5). The correlation with corn CCI and GPP anomalies in North Dakota was not significant at the emerged stage, while correlations with yield were significant in most counties in eastern North Dakota (r: 0.5–0.6), but not significant in western North Dakota.

The silking stage ranged from week 24 to week 30, or around late July to early August. The correlations between GPP and both CCI and yield anomalies increased going from the emerged stage to the silking stages in most areas (Figures 3A,5A). The correlations with county-level yield were significant and positively correlated in 30% of the counties with available yield and phenology data by the silking stage, with r-values ranging from 0.4 to 0.8. As with the emerged stage, spatial patterns were apparent, where more positive correlations were observed around and west of the 100th meridian and lower correlations were observed further east of the 100th meridian. As with the emerged stage, GPP correlations with yield tended to be higher in many counties compared to the spatially coarser CCI correlations in the respective states. For example, correlations between GPP and corn yield in most counties in North Dakota were significant, while the correlations between GPP and corn CCI were not. GPP correlations with yield tended to be more spatially homogeneous at the silking stage than at the emerged stage, particularly in North Dakota, South Dakota, Minnesota, Texas, and Kansas.

The timing of the crop maturity stage for corn occurred on weeks ranging from 30 to 38, or from around late July to late September. The GPP correlations with both CCI and yield anomalies continued to strengthen as the crop phenology progressed from the silking stage to the mature stage, again in areas around and west of the 100th meridian, although to a lesser extent than the stronger correlation increases observed between the emergent and silking stages (Figures 3A,5A). Significant correlations between GPP and corn yield were observed in 65% of counties, with r-values ranging from 0.4 to 0.9 by the mature stage. While correlations east of the 100th meridian generally increased, the correlations were often not significant. The exceptions east of the 100th meridian, where correlations with yield were significant and positive, were located in counties in Iowa and Indiana. At the mature stage, the county-level GPP correlations with yield were more aligned with the state-level CCI correlation pattern when compared to the emerged and silking stages.

#### 3.1.2. Soybeans

The emerged stage for soybeans occurred at weeks ranging from week 22 to 25, or around late June to early July. Correlations between emerged stage GPP anomalies and end of season soybean yield anomalies tended to be higher than with corn, particularly for counties within and around North Dakota, Minnesota, and Michigan and Iowa (r: 0.4–0.8) where correlations were significant and positive (Figure 3B). At the emerged stage, 13% of counties had positive and significant correlations between GPP and yield. The spatial distribution of GPP correlations with yield also tended to be more homogeneous for soybeans than with corn. However, there were similar correlation spatial patterns for corn and soybean yield anomalies during the emerged stage, where significant and negative r-values were observed in counties near the Missouri, Mississippi, and Ohio Rivers where irrigated production is dominant and the climate is more humid, and significant and positive correlations were observed around the 100th meridian. Figure 5B shows that correlations between GPP anomalies and CCI anomalies at the emerged stage were largely not significant, with the exception of Mississippi where a significant negative correlation was observed (r: −0.6). These results indicate that GPP anomalies derived from the L4C record often provide better insight into crop status in terms of expected yield anomalies than the state-level CCI in many counties, primarily located in North Dakota, Minnesota, and Kansas.

The blooming stage for soybeans occurred at weeks ranging from 28 to 32, or around mid-July to early August. As with corn’s second phenological stage, the correlations between GPP anomalies at the blooming stage and both CCI anomalies and yield anomalies increased, and more counties with significant correlations (33%) were observed (Figures 3B,5B). Generally, higher and significant correlations with yield (r: 0.6–0.9) were observed in most counties within CONUS during the blooming stage, with the exceptions again in counties close to the Mississippi and Ohio Rivers where correlations were often not significant. Significant correlations with CCI during the blooming stage were observed in South Dakota, Nebraska, and Kansas (r: 0.5–0.6).

The dropping leaves stage for soybeans ranged from week 36 to week 42, or around early September to mid-October. Correlations between GPP anomalies and yield anomalies continued to strengthen as the crop phenology progressed from this stage, with positive and significant r-values being observed in 60% of counties and with r-values ranging from 0.7 to 0.9 in many counties (Figure 3B). Nonsignificant correlations tended to occur in counties situated directly west of the Mississippi River, where production is dominantly irrigated. The GPP correlation pattern was similar for both CCI and yield. Figure 5B shows that the correlations between GPP anomalies and CCI anomalies at the dropping leaves stage were significant in most states, and the highest correlations were observed in Nebraska (r: 0.8), Kansas (r: 0.8), and South Dakota (r: 0.7). Soybean harvests generally occurred around 2 weeks after the dropping leaves stage and produced similar results.

#### 3.1.3. Barley and Spring Wheat

The emerged stage for both barley and spring wheat occurred between week 18 and week 22, or around early May to early June. Figure 4A shows that significant and positive correlations between GPP anomalies and barley yield were observed in counties situated around eastern Washington and western North Dakota (r: 0.5) and around western North Dakota and western South Dakota for spring wheat (r: 0.5–0.7). Significant and positive correlations between GPP and CCI at the emerged stage were observed in Montana (r: 0.4) and North Dakota (r: 0.4) for barley and in Montana (r: 0.4) and South Dakota (r: 0.6) for spring wheat (Figure 5C).

The heading stage predominantly occurred from week 25 to week 28 for barley and spring wheat, or around mid-June to late July. Correlations between GPP anomalies and yield anomalies for both barley (Figure 4A) and spring wheat (Figure 4B) increased between the emerged and heading stages, with significant and positive correlations (r: 0.6–0.9) occurring in counties around eastern Washington, eastern Montana, and western North Dakota, in addition to western South Dakota in the case of spring wheat. Similar to corn and soybeans, GPP correlations with yield for spring wheat and barley were often not significant further to the east, where correlations transitioned from significant to nonsignificant approximately at the center of North Dakota and South Dakota, which generally corresponds to the CONUS aridity gradient. Correlations between GPP anomalies and CCI anomalies were also higher at the heading stage than at the emerged stage for both cereal crops (Figures 5C,D), with all states except Minnesota having significant r-values for both barley and spring wheat (r: 0.4–0.6 and r: 0.6–0.9 for barley and spring wheat, respectively).

The coloring stage most often occurs during week 32 for both barley and spring wheat, or around early August. The GPP correlations with both crop yield (Figures 4A,B) and CCI (Figures 5C,D) anomalies became stronger as both cereal crops progressed to the coloring stage. Consistent with the earlier crop development stages, the highest correlations were observed in eastern Washington, eastern Montana, and western North Dakota, in addition to South Dakota in the case of spring wheat, with significant r-values ranging from 0.6 to 0.9 in many counties. Again, the GPP correlations with yield were weaker in the more humid eastern North Dakota and South Dakota regions. Correlations between GPP anomalies at the coloring stage and CCI for both barley and spring wheat were significant (r: 0.6–0.7) in all states with the exception of Minnesota.

### 3.2. Climate and Irrigation

**FIGURE 6:**
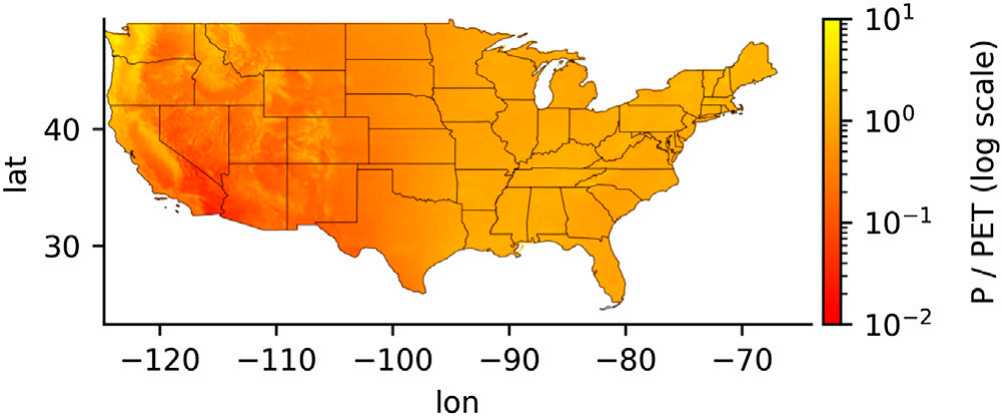
The Precipitation to Potential Evapotranspiration ratio (P/PET) for CONUS. Red colors indicate relatively more arid areas; yellow colors indicate relatively more humid areas. P/PET values at the 100th meridian ranged between 0.5 and 0.7, with values decreasing to the west and increasing to the east.

**FIGURE 7:**
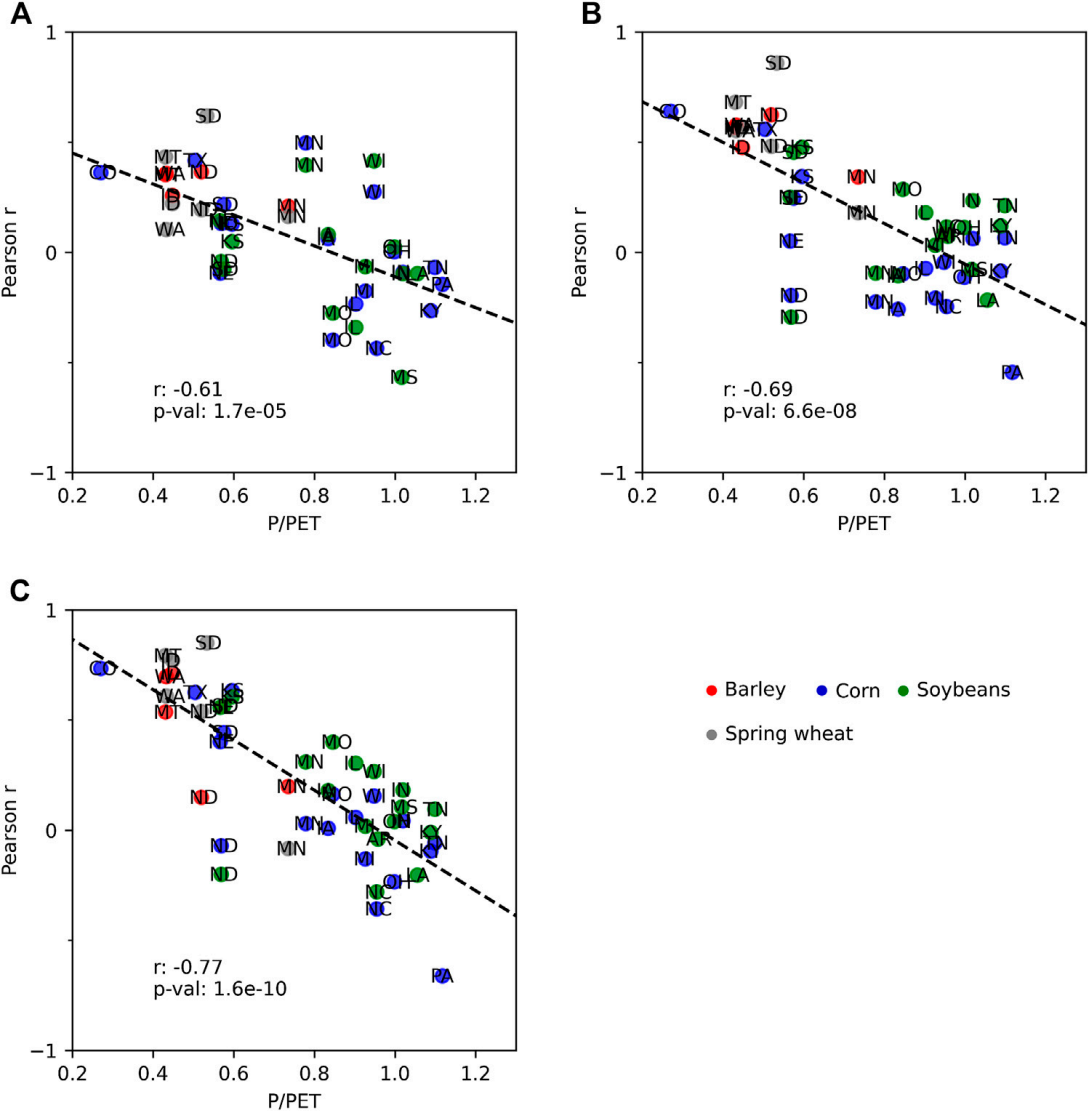
Changes in how the correlations between GPP anomalies and state‐level CCI anomalies vary with respect to the P/PET ratio at specific times during the growing season, where **(A)** shows week 26, **(B)** is week 30, and **(C)** is week 33. Each point represents the correlation of a particular crop grown in a particular state.

As described above, distinct spatial transitions in the correlations between L4C GPP and the CCI and yield anomalies were observed approximately west and east of the 100th meridian for all major crop types examined; the spatial pattern in these relationships generally follows the regional climate gradient between the more arid western and humid eastern portions of the CONUS domain (Figure 6). Areas where GPP tended to be a better indicator of crop status generally were in more arid regions where the average P/PET is less than 0.5. Figure 7 shows how the GPP correlations with CCI were generally weaker in areas with lower climate aridity, considering all states and crops, at the beginning, middle, and end of the growing season. While weaker correlations become more apparent as the season progresses, the relationship is significant across the entire growing season. Similar trends showing decreasing GPP correlations with crop yield in areas with less aridity were also found. However, considering irrigation along with climate aridity helped to explain some of the spatial differences in GPP correlations with crop yield.

**FIGURE 8:**
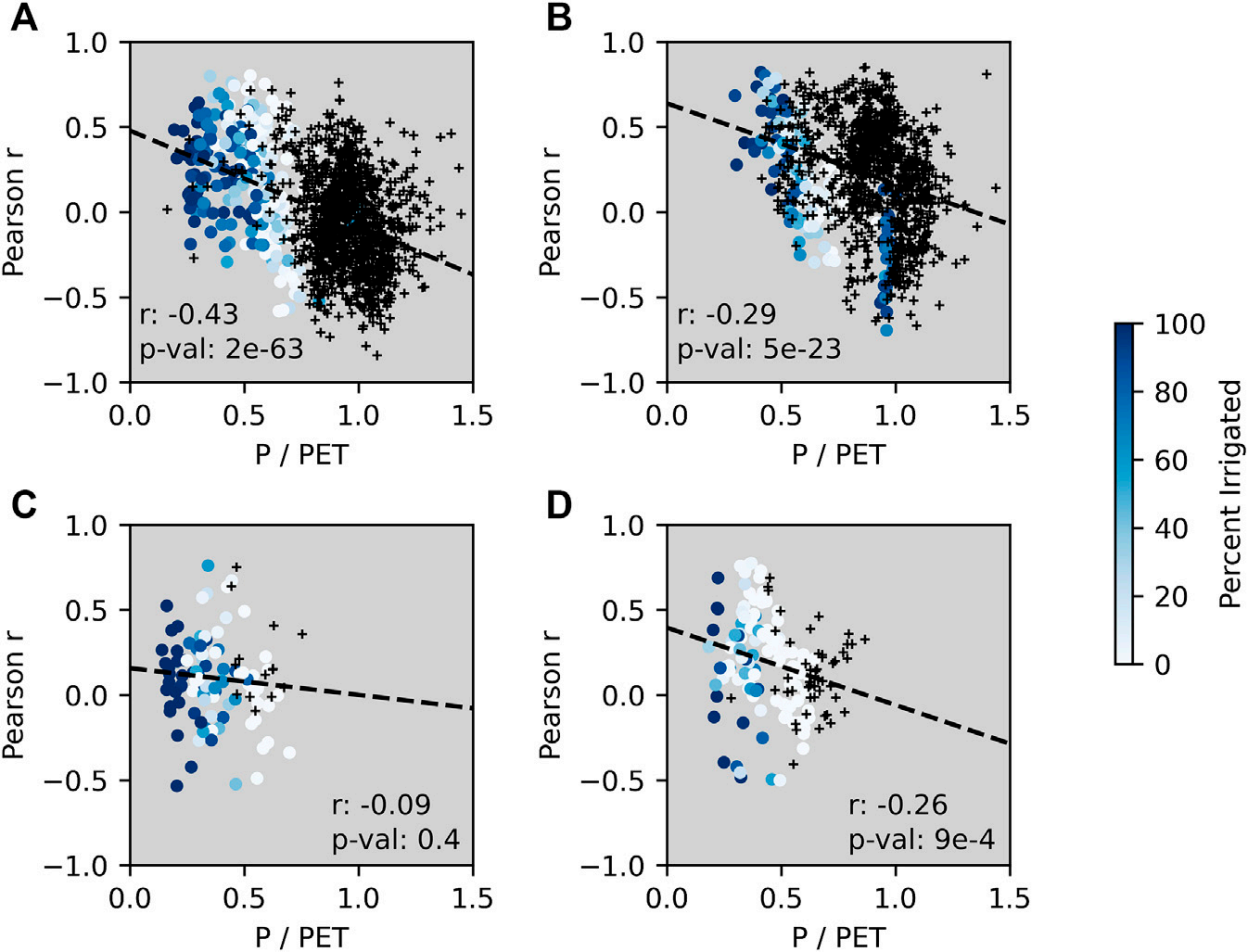
Changes in how the correlations between GPP anomalies and county‐level crop yield anomalies vary with respect to the P/PET ratio and percent irrigated production for corn **(A)**, soybeans **(B)**, barley **(C)**, and spring wheat **(D)** at around mid-season. Darker blues indicate greater percent irrigated production. The black crosses indicate that irrigation data was not available and so the percent irrigated production was not known. The dashed lines show the fitted linear regression with the corresponding r‐value and p‐value provided in the lower corner of each subplot.

**FIGURE 9:**
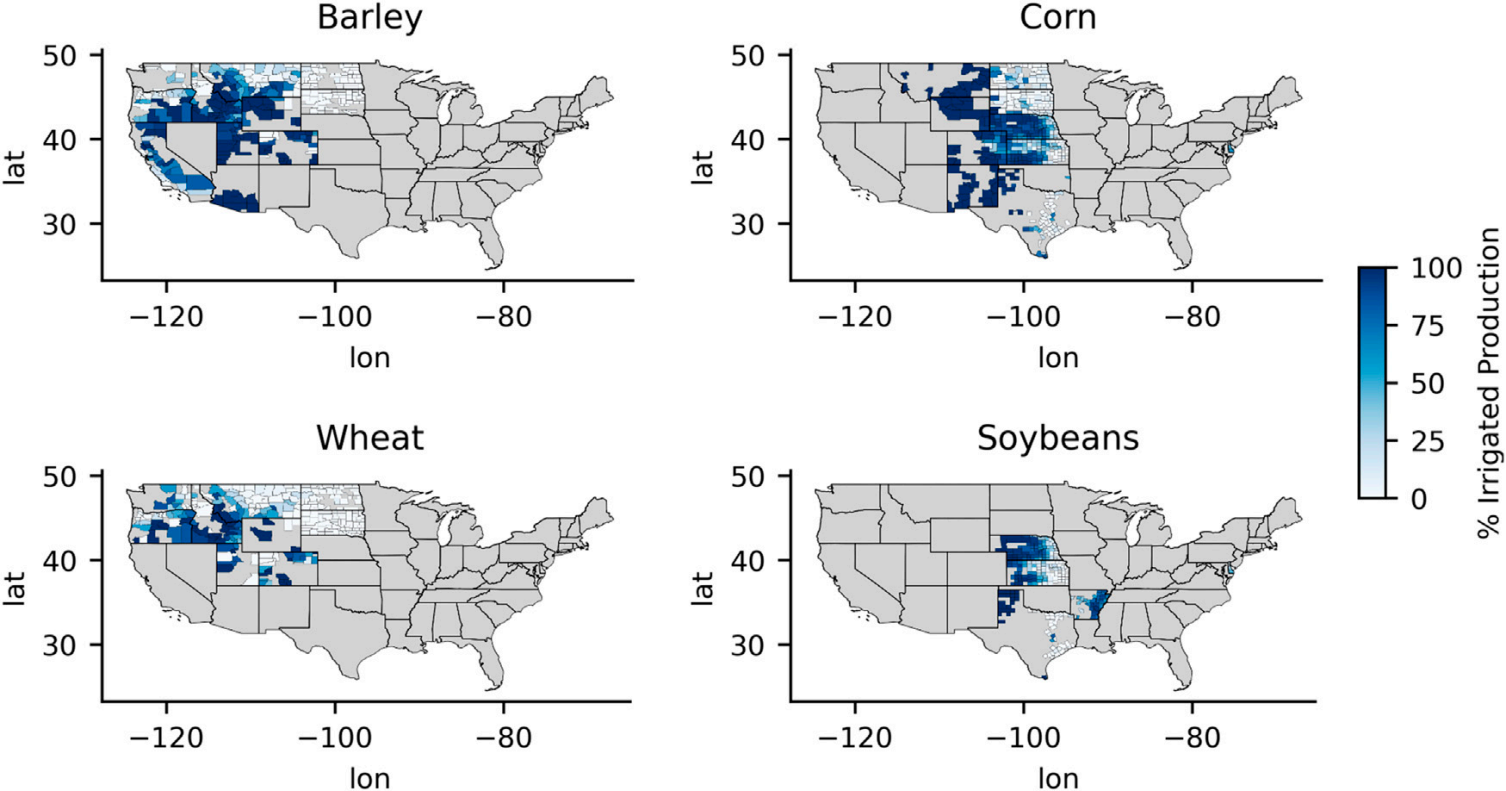
Percent of irrigated crop production at the county scale. Data: NASS.

Figure 8 shows how the GPP correlations with crop yield changed with respect to P/PET individually by crop type for all counties during the middle of the growing season; the figure also shows the percent of irrigated production for counties with available irrigation data. For reference, Figure 9 shows a map of the percent of irrigated production by county for the selected crop types examined. GPP tended to have the highest correlations with crop yield in counties with more arid climates where little or no irrigated production was present. The higher correlations observed in counties with higher irrigated production tended to be in more arid climates. Specifically for barley and spring wheat, irrigated production more often occurred in the most arid regions of the study area where agriculture would likely not be sustainable without irrigation. These arid areas include southern Idaho, Utah, and southern Arizona. The GPP correlation pattern was also more variable in these arid and heavily irrigated counties. However, the GPP correlations with barley and spring wheat yields were sharply weaker in less arid counties with no irrigation.

### 3.3. Environmental Drivers Affecting Level-4 Carbon Productivity

**FIGURE 10:**
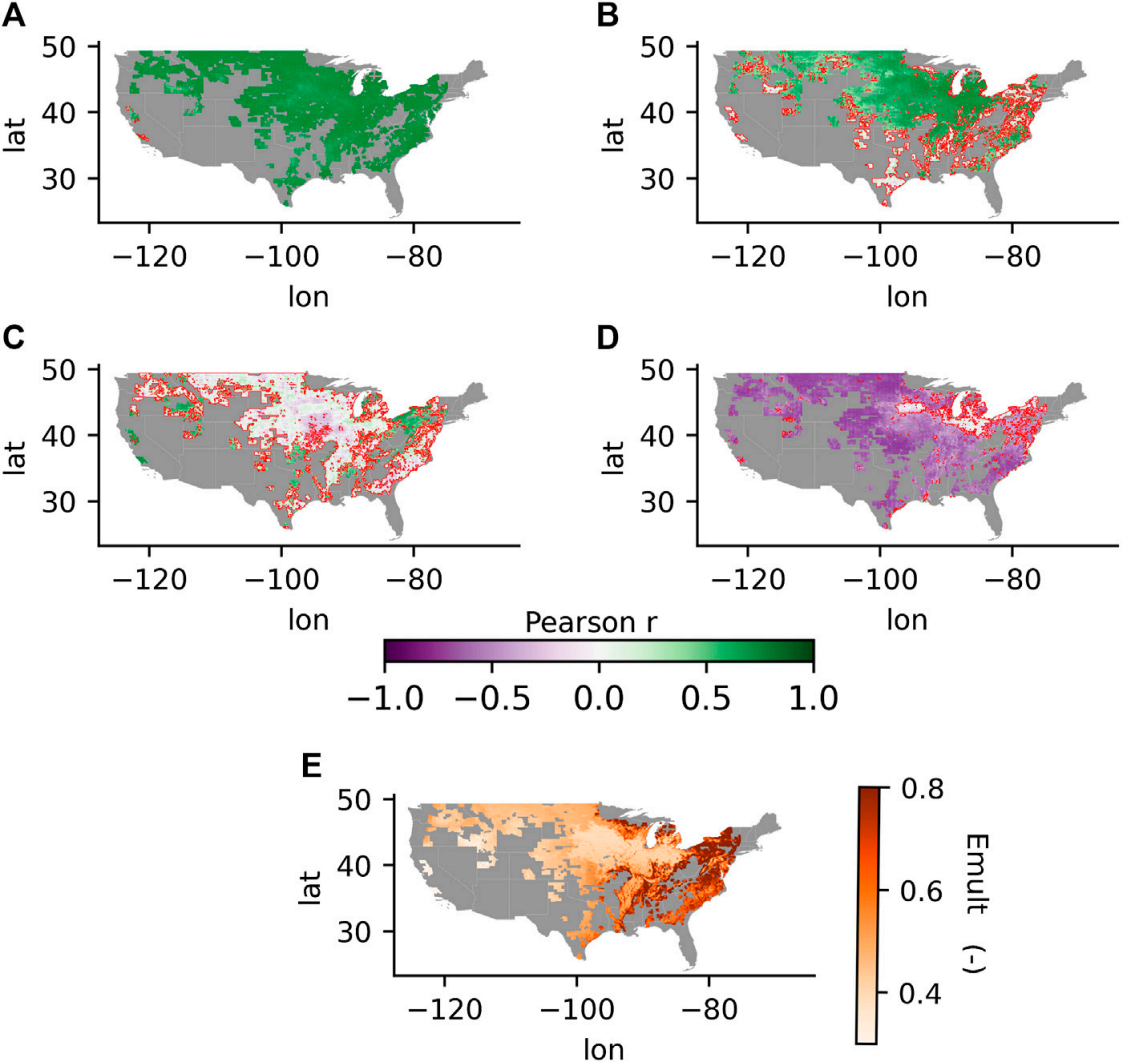
The correlation coefficients between GPP anomalies and the anomalies in **(A)** APAR, **(B)** Tmin, **(C)** RZSM, and **(D)** VPD for weeks 31‐33 in the growing season (mid‐way). Areas outlined in red show where correlations were not significant. **(E)** The mean growing season Emult term over the period of record.

We analyzed the spatial and temporal sensitivity of the L4C GPP record to APAR and the driving climatic variables. Figure 10 shows the spatial correlation pattern between anomalies in GPP and each driving variable calculated mid-season, including VPD, RZSM, Tmin, and APAR in counties where yield data was available. Figure 10 also shows the mean growing season $E_{\text{mult}}$ term derived over the period of record. VPD was strongly and inversely correlated with the standardized GPP anomaly over much of the domain, with the exception of areas east of the 100th meridian. The area where VPD had little influence on L4C GPP tended to expand as the season progressed. These areas where VPD had little to no influence generally corresponded with cultivated areas in the more humid climate regions where GPP showed lower correspondence with the CCI or crop yields. Correlations between GPP and RZSM were near zero during the emerged stage and increased slightly as the growing season progressed. RZSM had less influence on L4C GPP overall relative to the other climate drivers, although the correlation with soil moisture was stronger in the more arid regions. APAR was strongly and directly proportional to GPP across all weeks within the growing season. Tmin had correlations with GPP approaching unity in the same areas where the correlations between GPP and VPD, CCI, and crop yields were all relatively lower. The combined effect of the environmental restrictions on GPP (excluding APAR) as represented by the L4C $E_{\text{mult}}$ term (e.g., Eq. 2) was stronger in the more arid central and western portions of the CONUS domain, where GPP was approximately half (0.5) of the potential rate estimated for optimal conditions. A general longitudinal gradient toward weaker environmental restrictions and associated higher $E_{\text{mult}}$ levels occurs moving from the more arid western and central regions toward the more humid climate regions east of the 100th meridian, with the highest $E_{\text{mult}}$ levels (>0.8) occurring in the eastern CONUS. In these eastern regions, the L4C record indicates relatively little environmental restriction on productivity so that GPP is near the potential rate and variability in productivity is largely controlled by APAR.

## 4. Discussion

There was a marked improvement in the ability of the L4C-derived GPP to infer crop status as the season progressed. In terms of the two crop metrics used in this study, CCI and crop yield, Begueria and Maneta (2020) noted that CCI became more indicative of crop yield as the season progressed. The L4C results show a similar strengthening of the relationship between GPP and crop yield as the season progresses, where the accumulated carbon uptake in the first weeks following the emerged phase provides a small sample of crop response to the overall variability in environmental conditions accumulated over the course of the season. However, there is a limitation inherent to the L4C model that may also explain why lower correlations were observed at the beginning of the season, which is related to estimation of APAR when the canopy has yet to reach full coverage. Standing litter and soil background reflectance can introduce relatively higher error in the APAR calculations over sparse canopies (Smith et al., 2019). APAR is the primary driver of L4C GPP estimates, so that uncertainty in modeled APAR could potentially translate to greater uncertainty in GPP estimates when crops have just emerged.

Our study showed a clear difference in the ability of L4C GPP to capture crop status (both CCI and yield) in more arid regions vs. more humid climate regions. Inspection of the mean growing-season $E_{\text{mult}}$ term shows that, according to the L4C framework, GPP is operating under near-optimal conditions (i.e., high $E_{\text{mult}}$) in more humid areas. L4C GPP estimates in areas with high $E_{\text{mult}}$ will therefore be more constrained by APAR than the environmental constraints captured in the $E_{\text{mult}}$ term. For example, the L4C accounts for dry soil restrictions on GPP, but not the potential negative impacts contributed from wet soil events which occur more commonly in humid climate areas Li et al. (2019). The impact of wet soil events is highlighted by a major El Niño event that occurred in 2015–2016 which coincided with delayed planting due to intensive rainfall-driven flooding early in the season resulting in diminished yields in that year (Sadeghi et al., 2019). Negative impacts from this event would only partially be reflected in a reduction in fPAR indicated from the MODIS observational inputs to the L4C model and its associated effect on the GPP calculations; thus, the negative impact on yield would likely be underestimated from L4C relative to the CCI and reported crop yields. The impact of the El Niño event on GPP-yield correlations was observed by excluding 2016 from the analysis, which increased correlations from between 0.1 and 0.16 in several counties particularly around Louisiana and Alabama, although correlations in other counties were largely unchanged. However, differences in the response of vegetation to VPD and RZSM in drier and wetter climates may influence the accuracy of L4C GPP. In wetter climates, VPD has been found to be more important than RZSM for plant transpiration, while RZSM becomes a more dominant influence in drier climates (Novick et al., 2016). Figure 9 shows that, in the more humid areas where lower or negative GPP correlations were more widespread, VPD had the least influence on GPP estimates among the model drivers. While the influence of VPD on GPP is relatively stronger in arid regions, GPP is also influenced by RZSM, which may better reflect actual conditions. For example, Purdy et al. (2018) and Brust et al. (2019) found that the addition of SMAP RZSM into an ET model resulted in greater model ET improvements in drier areas than in wetter areas. The absolute estimated error (gC) for GPP is proportional to the size of the GPP flux, so that more productive regions show larger estimated RMSE (gC) than less productive drylands. However, the relative percent error (RMSE/GPP) is similar, indicating generally consistent model performance. While the addition of the RZSM control improves model performance in drylands, it has less impact in humid climates where VPD has stronger influence. In energy limited and/or wet climates, VPD may be directly proportional to GPP and only become inversely proportional to GPP for part of the year (e.g., summer drydown). Humid climates may also show less GPP correspondence due to less seasonal variation in productivity, since correlations are generally larger with greater seasonal amplitude in productivity. Differences in the impact of environmental controls on GPP with respect to the aridity index are not fully captured in the linear ramp functions shown in Eq. 3. For example, increased VPD or decreased RZSM may increase GPP in humid areas, while the L4C would indicate decreased GPP. This disagreement between actual and modeled GPP may partially explain the significant and negative correlations observed in humid areas. Additionally, increased contamination from cloud cover in humid areas introduces uncertainty in the MODIS fPAR estimates which then propagates into the GPP estimates in areas where APAR is the primary driver.

Aside from the model capability in different climates, crops grown in water-limited areas may have greater variability in response to meteorological anomalies than crops in energy-limited areas. For example, crop yields have higher correlations with PDSI, SPI, and SPEI in more arid regions (Rhee et al., 2010; Vicente-Serrano et al., 2012) and NDVI for cropland has higher correlations with SPEI in drier climates (Xu et al., 2018). The impact of VPD on GPP varies between wetter and drier climates for the same crop under similar soil moisture conditions, where crops in humid areas experience less reduction in GPP with higher VPD due to higher water use efficiency compared to that in more arid environments (Zhang et al., 2019). However, the sensitivity of crop production to climatic variability in more arid areas may be buffered by irrigation, which may partially explain why the L4C GPP anomalies were less consistent with county-level yield in arid regions dominated by irrigated production. An explicit representation of irrigation is lacking in the SMAP L4C and L4SM model framework so that the influence of irrigation on L4C GPP may only come through the MODIS fPAR observational inputs used to compute APAR. Situations where fPAR inputs and resulting APAR estimates better capture GPP may extend to other nonenvironmental stressors not captured by the $E_{\text{mult}}$ term such as nutrient deficiency or pests. As such, the L4C GPP better represents crop status in areas where environmental conditions are important limiting factors of the condition and yield of crops.

Quantifying the magnitude of decoupling between crop status and climatic conditions was difficult because irrigation data was absent in many counties. Further, there were many counties with similar climate and irrigation levels, but with different correlations between GPP and crop yield. In fact, GPP was highly correlated with crop yield in several (more arid) counties where production was almost completely irrigated. These discrepancies may reflect regional differences in irrigation method (e.g., sprinkler, flood, drip) and daily or seasonal water application regimes. Another source of error may be related to the relatively coarse (9-km) resolution of the RZSM estimates used as L4C inputs, which may not adequately represent soil moisture variability in heterogeneous croplands.

A major limitation in our analysis was that we did not account for cropping area of the specific study crops when calculating GPP anomalies. When comparing GPP anomalies to the state-level CCI anomalies, we used all available pixels corresponding with the associated cereal or broadleaf PFT instead of limiting GPP to the reported planted area of each specific crop. In other words, the broadleaf GPP estimated within a particular state was likely not produced solely from corn cultivation but was still included in the analysis. The situation was similar for yield, where GPP anomalies for cereal and broadleaf crop PFTs were not specifically attributed to a particular crop type within each county. While the NASS cropland data layer (CDL) provides tabulated annual estimates of planted acres by crop type, the CDL did not extend over the entire study period. The ability to include accurate cropping area into similar studies will therefore improve with a longer CDL record.

## 5. Summary and Implications

This study showed that the L4C GPP product provides a meaningful crop metric at relatively high spatial (county) and temporal (weekly) resolutions over cultivated lands across much of the CONUS domain. However, the application of the L4C GPP as a crop metric was better in arid regions with little irrigation, particularly after mid-season. In some areas, the correlations between GPP and county-level yield were significant as soon as the crop had emerged, indicating the potential of the L4C to provide a prediction of yield anomalies at the start of the season. The potential of the L4C to predict yield anomalies increases as the season progresses, as correlations were significant weeks or months prior to harvest. However, climate, land management practices, and phenological stage require careful consideration before making inferences regarding crop status using L4C.

## 6. Equations

$$\text{GPP}=\text{APAR}\cdot {ɛ}_{\text{max}}\cdot E_{\text{mult}}$$ (1)

where GPP is the gross primary production, canopy-APAR is the product of photosynthetically active radiation (PAR) and the fraction of PAR available to vegetation, ${ɛ}_{\text{max}}$ is the maximum light use efficiency (LUE) parameter defined for each plant function type (PFT) growing under optimal environmental conditions, and $E_{\text{mult}}$ is the estimated proportion of ${ɛ}_{\text{max}}$ occurring under the observed (suboptimal) environmental conditions.

$$E_{\text{mult}}=f_{\text{EC}}\left(\text{VPD}\right)f_{\text{EC}}\left(\text{TMIN}\right)f_{\text{EC}}\left(\text{RZSM}\right)f_{\text{EC}}\left(\text{FT}\right)$$ (2)

where $E_{\text{mult}}$ is the estimated proportion of $\varepsilon_{\text{max}}$ occurring under the observed (suboptimal) environmental conditions and is the product of dimensionless scalar multipliers representing the response of the different PFTs to vapor pressure deficit (VPD), minimum daily temperature (TMIN), and root zone soil moisture (RZSM). The freeze-thaw term (FT) is a binary term that represents frozen ground (0) or nonfrozen ground (1). The equation for calculating the $f_{\text{EC}}$ terms for each environmental constraint is given in Eq. 3.

$$f_{\text{EC}}\left(x\right)=\{\begin{array}{ll} 1 & \text{if}\;x\geq x_{\text{max}}\\ 0 & \text{if}\;x\leq x_{\text{max}}\\ \left(x-x_{\text{min}}\right)/\left(x_{\text{max}}-x_{\text{min}}\right) & \text{otherwise} \end{array}$$ (3)

where *x* is the observed VPD, TMIN, or RZSM, and $x_{\text{min}}$ and $x_{\text{max}}$ are parameters defined for the individual PFTs.

$${\text{GPPd}}_{k,i,j}={\text{GPP}}_{k,i,j}-f_{k,j}\left(i\right)$$ (4)

where GPPd is the detrended gross primary production at week *k* and year *i* in state *j*, GPP is the observed mean cumulative GPP at week *k* and year *i* in state *j*, and $f_{k}$ is a linear function of year *i* for GPP at week *k* in state *j*.

$$Z_{k,i,j}=\frac{{\text{GPPd}}_{k,i,j}-\mu_{k,j}}{\sigma_{k,j}}$$ (5)

where $Z_{k,i,j}$ is the z-score of GPP at week *k* and year *i* in state *j*, ${\text{GPP}}_{k,i,j}$ is the mean cumulative GPP for week *k* and year *i* in state *j*, $\mu_{k,j}$ is the mean detrended GPP for week *k* in state *j* over the period of record (near 0), and $\sigma_{k,j}$ is the standard deviation in GPPd for week *k* in state *j* over the period of record.

## Data Availability Statement

The raw data supporting the conclusions of this article will be made available by the authors, without undue reservation.

## Author Contributions

PW conducted the analysis and authored the paper. MM designed the study and edited the paper. JK provided L4C data, designed the study, and edited the paper. KE provided L4C data, designed the study, and edited the paper. SB provided crop condition index data and designed the study.

## Funding

This work was supported by the United States Department of Agriculture, USDA-NIFA research grant 2016-67026-25067, the NASA EPSCoR program research grant 80NSSC18M0025, and NASA projects NNX14AI50G and 80NSSC17K0115.

## Conflict of Interest

The authors declare that the research was conducted in the absence of any commercial or financial relationships that could be construed as a potential conflict of interest.

## References

[B1] Abatzoglou J. T. (2013). Development of gridded surface meteorological data for ecological applications and modelling. Int. J. Climatol. 33, 121–131. 10.1002/joc.3413

[B2] Amani M. Parsian S. MirMazloumi S. M. Aieneh O. (2016). Two new soil moisture indices based on the NIR-red triangle space of Landsat-8 data. Int. J. Appl. Earth Obs. Geoinf. 50, 176–186. 10.1016/j.jag.2016.03.018

[B3] Beer C. Reichstein M. Tomelleri E. Ciais P. Jung M. Carvalhais N. (2010). Terrestrial gross carbon dioxide uptake: global distribution and covariation with climate. Science 329, 834–838. 10.1126/science.1184984 20603496

[B4] Begueria S. Maneta M. M. (2020). Humans as sensors: qualitative crop condition survey data reveals spatiotemporal production patterns and allows early yield predictions. TBDProc. Natl. Acad. USA 117, 18317–18323. 10.1073/pnas.1917774117 PMC7414191 32675235

[B5] Brust C. Kimball J. S. Maneta M. P. Jencso K. He M. Reichle R. H. (2019). Using SMAP soil moisture to constrain MOD16 evapotranspiration estimates. Washington DC: American Geophysical Union.

[B6] Campbell J. E. Berry J. A. Seibt U. Smith S. J. Montzka S. A. Launois T. (2017). Large historical growth in global terrestrial gross primary production. Nature 544, 84–87. 10.1038/nature22030 28382993

[B7] Daryanto S. Wang L. Jacinthe P. A. (2017). Global synthesis of drought effects on cereal, legume, tuber and root crops production: a review. Agric. Water Manag. 179, 18–33. 10.1016/j.agwat.2016.04.022

[B8] Doughty R. Xiao X. Wu X. Zhang Y. Bajgain R. Zhou Y. (2018). Responses of gross primary production of grasslands and croplands under drought, pluvial, and irrigation conditions during 2010–2016, Oklahoma, USA. Agric. Water Manag. 204, 47–59. 10.1016/j.agwat.2018.04.001

[B9] Entekhabi B. D. Njoku E. G. Neill P. E. O. Kellogg K. H. Crow W. T. Edelstein W. N. (2010). The soil moisture active passive ( SMAP ) mission. Institue of Electrical and Electrongics Engineers 98, 704–716. 10.1109/jproc.2010.2043918

[B10] Fensholt R. Sandholt I. Stisen S. (2006). Evaluating MODIS, MERIS, and VEGETATION vegetation indices using *in situ* measurements in a semiarid environment. IEEE Trans. Geosci. Rem. Sens. 44, 1774–1786. 10.1109/TGRS.2006.875940

[B11] Friedl M. A. Sulla-Menashe D. Tan B. Schneider A. Ramankutty N. Sibley A. (2010). MODIS Collection 5 global land cover: algorithm refinements and characterization of new datasets. Remote Sensing of Environment 114, 168–182. 10.1016/j.rse.2009.08.016

[B12] Gelaro R. McCarty W. Suárez M. J. Todling R. Molod A. Takacs L. (2017). The modern-era retrospective analysis for research and applications, version 2 (MERRA-2). J. Clim. 30, 5419–5454. 10.1175/JCLI-D-16-0758.1 32020988 PMC6999672

[B13] Gower S. T. Kucharik C. J. Norman J. M. (1999). Direct and indirect estimation of leaf area index, f(APAR), and net primary production of terrestrial ecosystems. Remote Sensing of Environment 70, 29–51. 10.1016/S0034-4257(99)00056-5

[B14] Gu Y. Hunt E. Wardlow B. Basara J. B. Brown J. F. Verdin J. P. (2008). Evaluation of MODIS NDVI and NDWI for vegetation drought monitoring using Oklahoma Mesonet soil moisture data. Geophys. Res. Lett. 35, 1–5. 10.1029/2008GL035772

[B15] Guan K. Wu J. Kimball J. S. Anderson M. C. Frolking S. Li B. (2017). The shared and unique values of optical, fluorescence, thermal and microwave satellite data for estimating large-scale crop yields. Remote Sens. Environ. 199, 333–349. 10.1016/j.rse.2017.06.043

[B16] Guanter L. Zhang Y. Jung M. Joiner J. Voigt M. Berry J. A. (2014). Global and time-resolved monitoring of crop photosynthesis with chlorophyll fluorescence. Proc. Nat.Acad.Sci. 111, E1327–E1333. 10.1073/pnas.1320008111 24706867 PMC3986187

[B17] Gudmundsson L. Bremnes J. B. Haugen J. E. Engen-Skaugen T. (2012). Technical Note: downscaling RCM precipitation to the station scale using statistical transformations - a comparison of methods. Hydrol. Earth Syst. Sci. 16, 3383–3390. 10.5194/hess-16-3383-2012

[B18] He L. Chen J. M. Liu J. Mo G. Bélair S. Zheng T. (2014). Optimization of water uptake and photosynthetic parameters in an ecosystem model using tower flux data. Ecol. Model. 294, 94–104. 10.1016/j.ecolmodel.2014.09.019

[B19] Hilker T. Coops N. C. Wulder M. A. Black T. A. Guy R. D. (2008). The use of remote sensing in light use efficiency based models of gross primary production: a review of current status and future requirements. Sci. Total Environ. 404, 411–423. 10.1016/j.scitotenv.2007.11.007 18063011

[B20] Hobbins M. T. Wood A. McEvoy D. J. Huntington J. L. Morton C. Anderson M. (2016). The evaporative demand drought index. Part I: linking drought evolution to variations in evaporative demand. J. Hydrometeorol. 17, 1745–1761. 10.1175/JHM-D-15-0121.1

[B21] Ji L. Peters A. J. (2003). Assessing vegetation response to drought in the northern Great Plains using vegetation and drought indices. Remote Sensing of Environment 87, 85–98. 10.1016/S0034-4257(03)00174-3

[B22] Jones L. A. Kimball J. S. Reichle R. H. Madani N. Glassy J. Ardizzone J. V. (2017). The SMAP level 4 carbon product for monitoring ecosystem land-atmosphere CO2 exchange. IEEE Trans. Geosci. Rem. Sens. 55, 6517–6532. 10.1109/TGRS.2017.2729343

[B23] Klink K. Wiersma J. J. Crawford C. J. Stuthman D. D. (2014). Impacts of temperature and precipitation variability in the Northern Plains of the United States and Canada on the productivity of spring barley and oat. Int. J. Climatol. 34, 2805–2818. 10.1002/joc.3877

[B24] Lehecka G. V. (2014). The Value of USDA crop progress and condition information: reactions of corn and soybean futures markets. J. Agric. Resour. Econ. 39, 88–105. 10.22004/ag.econ.168261

[B25] Li Y. Guan K. Schnitkey G. D. DeLucia E. Peng B. (2019). Excessive rainfall leads to maize yield loss of a comparable magnitude to extreme drought in the United States. Global Change Biol. 25, 2325–2337. 10.1111/gcb.14628 PMC6850578 31033107

[B26] Mallick K. Bhattacharya B. K. Patel N. (2009). Estimating volumetric surface moisture content for cropped soils using a soil wetness index based on surface temperature and NDVI. Agric. For. Meteorol. 149, 1327–1342. 10.1016/j.agrformet.2009.03.004

[B27] McKee T. B. Doesken N. J. Kleist J. (1993). “The relationship of drought frequency and duration to time scales,” in Proceedings of the Eighth Conference on Applied Climatology, California (American Meteorological Society), 179–184 *.*

[B28] Meyer S. J. Hubbard K. G. Wilhite D. A. (1991). The relationship of climatic indices and variables to corn (maize) yields: a principal components analysis. Agric. For. Meteorol. 55, 59–84. 10.1016/0168-1923(91)90022-I

[B29] Mishra A. K. Singh V. P. (2010). A review of drought concepts. J. Hydrol. 391, 202–216. 10.1016/j.jhydrol.2010.07.012

[B30] Monteith J. L. (1972). Solar radiation and productivity in tropical ecosystems. J. Appl. Ecol. 9, 747. 10.2307/2401901

[B31] Nemani R. Pierce L. Running S. Goward S. (1993). Developing satellite-derived estimates of surface moisture status. J. Appl. Meteorol. 32, 548–557. 10.1175/1520-0450(1993)032<0548:DSDEOS>2.0.CO;2

[B32] Njoku E. G. Entekhabi D. (1996). Passive microwave remote sensing of soil moisture. J. Hydrol. 184, 101–129. 10.1016/0022-1694(95)02970-2

[B33] Novick K. A. Ficklin D. L. Stoy P. C. Williams C. A. Bohrer G. Oishi A. C. (2016). The increasing importance of atmospheric demand for ecosystem water and carbon fluxes. Nat. Clim. Change 6, 1023–1027. 10.1038/nclimate3114

[B34] Palmer W. C. (1965). Meteorological drought [Dataset], U.S. Department of Commerce, Weather Bureau.

[B35] Pastor J. Post W. M. (1986). Influence of climate, soil moisture, and succession on forest carbon and nitrogen cycles. Biogeochemistry 2, 3–27. 10.1007/BF02186962

[B36] Peña-Gallardo M. Vicente-Serrano S. M. Quiring S. Svoboda M. Hannaford J. Tomas-Burguera M. (2019). Response of crop yield to different time-scales of drought in the United States: spatio-temporal patterns and climatic and environmental drivers. Agric. For. Meteorol. 264, 40–55. 10.1016/j.agrformet.2018.09.019

[B37] Pingali P. L. (2012). Green revolution: impacts, limits, andthe path ahead. Proceedings of the National Acad. Sci. USA 109, 12302–12308. 10.1073/pnas.0912953109 PMC3411969 22826253

[B38] Purdy A. J. Fisher J. B. Goulden M. L. Colliander A. Halverson G. Tu K. (2018). SMAP soil moisture improves global evapotranspiration. Remote Sens. Environ. 219, 1–14. 10.1016/j.rse.2018.09.023

[B39] Rahman A. F. Sims D. A. Cordova V. D. El-Masri B. Z. (2005). Potential of MODIS EVI and surface temperature for directly estimating per-pixel ecosystem C fluxes. Geophys. Res. Lett. 32, 1–4. 10.1029/2005GL024127

[B40] Reichle R. De Lannoy G. Liu Q. Ardizzone J. Kimball J. Koster R. (2016). “SMAP level 4 surface and root zone soil moisture,” in IEEE international geoscience and remote sensing symposium (IGARSS), China, July 10–15, 2016 (IEEE), 136–138. 10.1109/IGARSS.2016.7729026

[B41] Reichle R. H. De Lannoy G. J. Liu Q. Ardizzone J. V. Colliander A. Conaty A. (2017). Assessment of the SMAP Level-4 surface and root-zone soil moisture product using *in situ* measurements. J. Hydrometeorol. 18, 2621–2645. 10.1175/JHM-D-17-0063.1 PMC6196324 30364509

[B42] Rhee J. Im J. Carbone G. J. (2010). Monitoring agricultural drought for arid and humid regions using multi-sensor remote sensing data. Remote Sensing of Environment 114, 2875–2887. 10.1016/j.rse.2010.07.005

[B43] Rouse J. W. Hass H. R. Jr. Deering J. A. Harland J. C. (1974). Monitoring the vernal advancement and retrogradation (greenwave effect) of natural vegetation. Greenbelt, MD: NASA Goddard Space Flight Center.

[B44] Running S. W. Nemani R. R. Heinsch F. A. Zhao M. Reeves M. Hashimoto H. (2004). A continuous satellite-derived measure of global terrestrial primary production. BioScience 54, 547. 10.1641/0006-3568(2004)054[0547:ACSMOG]2.0.CO;2

[B45] Sadeghi S. T. Tootle G. Elliott E. Lakshmi V. Therrell M. Kalra A. (2019). Implications of the 2015-2016 El Niño on coastal Mississippi-Alabama streamflow and agriculture. Hydrology 6, 1–10. 10.3390/HYDROLOGY6040096

[B46] Sanders K. T. Masri S. F. (2016). The energy-water agriculture nexus: the past, present and future of holistic resource management via remote sensing technologies. J. Clean. Prod. 117, 73–88. 10.1016/j.jclepro.2016.01.034

[B47] Seneviratne S. I. Corti T. Davin E. L. Hirschi M. Jaeger E. B. Lehner I. (2010). Investigating soil moisture-climate interactions in a changing climate: a review. Earth Sci. Rev. 99, 125–161. 10.1016/j.earscirev.2010.02.004

[B48] Smith W. K. Dannenberg M. P. Yan D. Herrmann S. Barnes M. L. Barron-Gafford G. A. (2019). Remote sensing of dryland ecosystem structure and function: progress, challenges, and opportunities. Remote Sensing of Environment 233, 111401. 10.1016/j.rse.2019.111401

[B49] Tucker C. J. (1979). Red and photographic infrared linear combinations for monitoring vegetation. Remote Sensing of Environment 8, 127–150. 10.1016/0034-4257(79)90013-0

[B50] Vicente-Serrano S. M. Beguería S. López-Moreno J. I. (2010). A multiscalar drought index sensitive to global warming: the standardized precipitation evapotranspiration index. J. Clim. 23, 1696–1718. 10.1175/2009JCLI2909.1

[B51] Vicente-Serrano S. M. Beguería S. Lorenzo-Lacruz J. Camarero J. J. López-Moreno J. I. Azorin-Molina C. (2012). Performance of drought indices for ecological, agricultural, and hydrological applications. Earth Interact. 16, 1–27. 10.1175/2012EI000434.1

[B52] Wurster P. Maneta M. Begueria S. Cobourn K. Maxwell B. Silverman N. (2020). Characterizing the impact of climatic and price anomalies on agrosystems in the northwest United States. Agric. For. Meteorol. 280, 107778. 10.1016/j.agrformet.2019.107778

[B53] Xu H.-j. Wang X.-p. Zhao C.-y. Yang X.-m. (2018). Diverse responses of vegetation growth to meteorological drought across climate zones and land biomes in northern China from 1981 to 2014. Agric. For. Meteorol. 262, 1–13. 10.1016/j.agrformet.2018.06.027

[B54] Zhang Q. Ficklin D. L. Manzoni S. Wang L. Way D. Phillips R. P. (2019). Response of ecosystem intrinsic water use efficiency and gross primary productivity to rising vapor pressure deficit. Environ. Res. Lett. 14, 074023. 10.1088/1748-9326/ab2603

[B55] Zipper S. C. Qiu J. Kucharik C. J. (2016). Drought effects on US maize and soybean production: spatiotemporal patterns and historical changes. Environ. Res. Lett. 11, 94021. 10.1088/1748-9326/11/9/094021

